# What are the most important infectious diseases among those ≥65 years: a comprehensive analysis on notifiable diseases, Norway, 1993–2011

**DOI:** 10.1186/1471-2334-14-57

**Published:** 2014-02-04

**Authors:** Anneke Steens, Hanne-Merete Eriksen, Hans Blystad

**Affiliations:** 1 Norwegian Institute of Public Health, Oslo, Norway; 2 European Programme for Intervention Epidemiology Training (EPIET), European Centre for Disease Prevention and Control (ECDC), Stockholm, Sweden

## Abstract

**Background:**

As the population ages, the burden on the healthcare system might increase and require changed public health priorities. As infections are often more severe at older age, we rank notifiable infectious diseases (ID) and describe trends of ID among the general population aged ≥65 years in Norway in order to inform public health priorities for the aging population.

**Methods:**

We included all eligible cases of the 58 IDs notified between 1993 and 2011 (n = 223,758; 12% ≥65 years) and determined annual notification rates as the number of notified cases divided by the number of inhabitants of the corresponding year. We ranked diseases using their average annual notification rate for 2007–2011. Trends in notification rates from 1993 onwards were determined with a non-parametric test for trend. Using notification rate ratios (NRR), we compared results in those aged ≥65 years to those aged 20–64 years.

**Results:**

Invasive pneumococcal disease was the most common ID among the population ≥65 years (notification rate 58/100,000), followed by pertussis (54/100,000) and campylobacteriosis (30/100,000). Most ID notification rates did not change over time, though the notification rate of symptomatic MRSA infections increased from 1/100,000 in 1995 (first year of notification) to 14/100,000 in 2011.

Overall, fewer cases were notified among the population ≥65 years compared to 20–64 year olds (NRR = 0.73). The NRR of each of the invasive bacterial diseases and antibiotic-resistant infections were above 1.5 (i.e. more common in ≥65), while the NRR of each food- and waterborne disease, blood-borne disease/STI and (non-invasive) vaccine preventable disease was below 1.

**Conclusions:**

Based on our results, we emphasise the importance of focusing public health efforts for those ≥65 years on preventing invasive bacterial infections. This can be achieved by increasing pneumococcal and influenza vaccine uptake, and risk communication including encouraging those aged ≥65 years and their caretakers to seek healthcare at signs of systemic infection. Furthermore, good compliance to infection control measures, screening of the at-risk population, and careful use of antibiotics may prevent further increase in antibiotic-resistant infections.

## Background

In almost all Western countries, the proportion of the population aged 65 years or older (≥65 years) is increasing. In Norway, a 10% increase in the population ≥65 years has been seen over the last 25 years, reaching almost 750,000 in 2011 (15% of the five million Norwegian inhabitants). The increase is expected to continue due to longer survival and the aging post-war baby boom generation [1]. With older age, infections are often more severe due to factors such as the presence of multiple underlying medical conditions, weakened immune system (immunosenescence), concurrent use of different drugs (polypharmacy), delayed diagnosis, and/or delayed or diminished response to therapy [2-5]. Living in close proximity, such as in elderly homes and nursing homes, facilitates transmission of infectious agents among the older population [5,6]. An aging population may therefore increase the burden on the healthcare system. Furthermore, as the mortality rate resulting from infectious diseases (ID) has been increasing since the 1990s specifically for this older population [7], adaptations in public health priorities might be needed.

Although much research has been done for those ≥65 years on specific IDs, like influenza [8], urinary tract infection [9], bacterial meningitis [10], group B streptococcal infection [11] and gastroenteritis [12], and on vulnerable groups like long-term care residents [12,13], less is known about IDs in the general population ≥65 years. Furthermore, there is only limited information available on time trends for different IDs. This information is especially important with shifting demographics, age-specific behavioural changes and changes in vaccine policy, to assist policymakers to choose appropriate public health responses.

As Norway has had a nationwide surveillance system in place since the 1970s [14], we were able to describe IDs and their trends among the general population ≥65 years in Norway from 1993 to 2011. The results were then compared to results of younger age groups. This study can be used to inform public health priorities for the aging population.

## Methods

### Design and study population

We used nationwide data from the Norwegian Surveillance System for Communicable Diseases (MSIS) [14]. The study population includes all Norwegian inhabitants and the study period includes 1993 through 2011.

### Data sources

MSIS has been in place since 1975 and is based at the Norwegian Institute of Public Health (NIPH). Clinicians and laboratories are obliged by law to report each case of the 58 notifiable IDs to MSIS. Reports from clinicians and laboratories on each case are matched by personal identification number (see Additional file 1). According to the MSIS regulations, the NIPH does not require ethical approval for the use of notified data for this type of study.

As few changes in case definitions have been implemented since 1993, we included all cases with a testing date between January 1st 1993 and December 31st 2011, who were registered before January 30th 2012. We only included symptomatic cases, except for methicillin-resistant *Staphylococcus areus* (MRSA), vancomycin-resistant enterococci (VRE) and chronic infections (hepatitis B and C, HIV, syphilis and tuberculosis), for which we also included asymptomatic cases. We excluded IDs for which no cases among persons ≥65 years were notified during the study period (anthrax, diphtheria, echinococcal disease, epidemic typhus, haemorrhagic fevers, leprosy, measles, plague, poliomyelitis, rabies, relapsing fevers, SARS, smallpox, trichinosis and yellow fever). Furthermore, we excluded influenza and genital chlamydia because only limited individual data is reported to MSIS.

Publicly available demographic data for Norway (number of inhabitants at January 1st of each corresponding year [15]) were used as the denominator.

### Data analysis

Data were analysed in Stata 12 (Stata Corporation, USA) and Excel (version 2010). We determined annual notification rates as the number of notified cases divided by the number of inhabitants of the corresponding year, expressed per 100,000 persons. To rank diseases based on their occurrence in recent years, we used data over the period 2007–2011 and calculated the average annual notification rate, in order to correct for yearly fluctuations. To investigate general patterns in IDs, we determined the percentage of IDs per predefined ID category (see Table 1). The category ‘invasive bacterial diseases’ was defined based on isolation of bacteria from blood or other sterile site. The vaccine preventable diseases category only comprised diseases of the childhood immunisation program, excluding the invasive bacterial diseases.

**Table 1:** Age-specific average annual frequency and notification rates of IDs in Norway in 2007 to 2011

Notifiable infectious disease^*^	**Average annual frequency**		**Average annual notification rate (range)/100,000 inhabitants**					**NRR **^**7 **^**(range)**
	**Total**	**≥65 years**	**Total**	**<5 years**	**5-19 years**	**20-64 years**	**≥65 years**	**65+/20-64**
Overall	15870	1792	332 (307–362)	330 (285–407)	330 (276–399)	351 (312–411)	253 (236–280)	0.7 (0.6-0.9)
*Invasive bacterial diseases (IBD)*								
Pneumococcal IBD	818	411	17.1 (14.9-20.4)	13.0 (9.1-19.7)	2.3 (1.2-3.1)	12.1 (10.1-14.9)	58.1 (51.6-66.4)	4.8 (4.4-5.1)
Group B Streptococcal IBD	178	78	3.7 (3.4 -3.9)	14.1 (12.3-15.9)	0.2 (0.0-0.4)	2.0 (1.8-2.2)	11.0 (9.8-12.5)	5.6 (4.5-7.6)
Group A Streptococcal IBD	163	68	3.4 (2.8-3.6)	2.6 (1.0)	0.7 (0.4-0.9)	2.8 (2.1-3.2)	9.5 (8.8-10.7)	3.4 (2.7-4.5)
*Haemophilus influenzae* IBD	81	40	1.7 (1.5-1.8)	2.2 (1.4-3.4)	0.4 (0.2-0.5)	1.1 (0.8-1.4)	5.7 (4.4-7.0)	5.6 (3.5-8.9)
Meningococcal IBD	37	5.8	0.8 (0.6-0.9)	3.2 (2.3-4.1)	1.3 (0.9-2.0)	0.3 (0.3-0.4)	0.8 (0.3-1.1)	2.4 (1.0-3.6)
*Food- and waterborne diseases*^1^								
Campylobacteriosis	2839	217	59.2 (55.0-60.9)	62.4 (47.7-77.7)	36.2 (31.1-41.6)	73.4 (70.5-75.6)	30.5 (25.1-38.3)	0.4 (0.4-0.5)
Salmonellosis	1485	125	31.0 (26.0-40.8)	48.2 (36.9-56.9)	22.4 (18.1-30.0)	35.3 (28.0-47.5)	17.6 (12.3-21.8)	0.5 (0.4-0.7)
Listeriosis	31	20	0.7 (0.4-1.0)	0.3 (0.0-0.7)	0.0 (0.0-0.0)	0.4 (0.1-0.7)	2.8 (2.3-4.2)	15.6 (3.8-37.5)
Enteropathogenic E. coli-enteritis ^2^	272	9.2	5.6 (2.3-9.3)	61.1 (15.2-115.9)	2.2 (0.8-3.2)	2.0 (1.8-2.2)	1.3 (0.4-2.2)	0.6 (0.2-1.0)
Giardiasis	192	9.0	4.0 (3.6-5.0)	7.9 (4.8-10.1)	3.4 (2.8-3.7)	4.5 (3.9-5.8)	1.3 (0.8-1.9)	0.3 (0.2-0.3)
Shigellosis	144	6.4	3.0 (2.7-3.3)	3.7 (1.9-5.5)	1.8 (1.4-2.4)	3.8 (3.3-4.7)	0.90 (0.3-1.4)	0.2 (0.1-0.4)
Paratyphoid fever	16	1.0	0.3 (0.2-0.4)	0.3 (0.0-0.7)	0.3 (0.0-0.9)	0.4 (0.2-0.5)	0.1 (0.0-0.4)	0.4 (0.0-1.1)
Yersiniosis	59	2.8	1.2 (1.1-1.5)	3.4 (2.2-4.4)	1.0 (0.7-1.3)	1.3 (1.0-1.7)	0.4 (0.0-0.7)	0.3 (0. 0–0.6)
Typhoid fever	17	0.2	0.4 (0.2-0.6)	0.6 (0.3-1.7)	0.5 (0.4-0.5)	0.4 (0.2-0.7)	0.0 (0.0-0.1)	0.0 (0.0-0.2)
*Vaccine preventable diseases*								
Pertussis	4328	383	90.4 (66.3-115.5)	56.3 (42.8-63.8)	211.5 (162.0-277.6)	63.4 (44.0-82.1)	54.2 (34.8-70.4)	0.9 (0.7-1.0)
Mumps ^3^	16	1.4	0.3 (0.2-0.5)	0.1 (0.0-0.6)	0.3 (0.1-1.0)	0.4 (0.3-0.5)	0.2 (0.1-0.3)	0.6 (0.3-1.0)
Tetanus	1.0	1.0	0.0 (0.0-0.0)	0.0 (0.0-0.0)	0.0 (0.0-0.0)	0.0 (0.0-0.0)	0.1 (0.0-0.3)	∞ (∞)
*Antibiotic-resistant bacteria*								
MRSA infection ^3, 4^	420	92	8.7 (7.3-11.4)	11.0 (8.8-13.3)	6.5 (5.1-9.4)	8.2 (6.6-11.4)	13 (11.4-16.1)	1.6 (1.2-2.1)
MRSA carriage ^3, 4^	384	82	8.0 (5.6-9.9)	16.3 (8.6-21.4)	4.6 (1.5-6.6)	7.3 (4.9-9.2)	12.2 (10.6-14.8)	1.7 (1.2-2.7)
Vancomycin-resistant enterococci ^3^	51	33	1.0 (0.1-4.3)	0.1 (0.1-0.3)	0.1 (0.0-0.4)	0.6 (0.1-2.3)	4.5 (0.6-19.4)	6.5 (4.0-8.6)
Penicillin-resistant pneumococci ^3^	11	1.4	0.2 (0.0-0.5)	1.1 (0.0-3.5)	0.1 (0.0-0.2)	0.2 (0.0-0.4)	0.2 (0.1-0.3)	1.7 (0.4-4.0)
*Zoonosis/vector-borne diseases*								
Lyme borreliosis ^3^	297	57	6.2 (5.1-7.3)	5.6 (4.6-6.2)	8.9 (6.3-10.9)	4.9 (3.6-6.4)	8.1 (6.3-9.8)	1.7 (1.5-1.9)
Tularaemia	66	10	1.4 (0.3-3.6)	0.7 (0.0-2.3)	1.0 (0.2-2.8)	1.5 (0.3-3.9)	1.4 (0.1-3.8)	0.8 (0.4-1.0)
HFRS ^5^/Nephropathia epidemica	40	4.6	0.8 (0.4-1.6)	0.1 (0.0-0.3)	0.3 (0.1-0.6)	1.2 (0.6-2.3)	0.7 (0.3-1.2)	0.7 (0.2-1.0)
Malaria	32	1.0	0.7 (0.6-0.8)	0.2 (0.0-0.3)	0.2 (0.1-0.3)	1.0 (0.9-1.2)	0.1 (0.0-0.4)	0.1 (0.0-0.4)
*Blood-borne/STI*								
Hepatitis C ^3, 6^	2270	36	47.2 (33.7-70.5)	2.3 (1.0-4.8)	5.1 (4.0-7.0)	76.1 (53.6-114.1)	5.0 (4.6-5.8)	0.1 (0.1-0.1)
Hepatitis B (chronic carriage)	693	13	14.4 (14.3-17.4)	0.7 (0.0-1.4)	8.5 (6.4-11.0)	20.9 (15.7-24.9)	1.8 (1.0-2.3)	0.1 (0.1-0.1)
Gonorrhoea	266	4.2	5.5 (4.6-6.3)	0.1 (0.0-0.3)	1.4 (0.9-1.6)	8.7 (7.4-9.8)	0.6 (0.1-1.2)	0.1 (0.0-0.1)
Hepatitis A	36	3.0	0.7 (0.4-1.0)	1.1 (0.7-2.3)	0.9 (0.6-1.4)	0.7 (0.3-1.1)	0.4 (0.1-1.0)	0.6 (0.4-1.4)
HIV infection	256	2.6	5.3 (4.9-5.9)	0.9 (0.0-2.1)	0.6 (0.4-0.9)	8.6 (8.0-9.5)	0.4 (0.3-0.6)	0.0 (0.0-0.1)
AIDS	33	2.2	0.7 (0.4-0.9)	0.0 (0.0-0.0)	0.0 (0.0-0.2)	1.0 (0.6-1.4)	0.3 (0.0-1.0)	0.3 (0.0-1.0)
Hepatitis B (acute infection)	71	1.4	1.5 (0.6-2.6)	0.1 (0.0-0.3)	0.6 (0.1-1.3)	2.2 (0.9-3.8)	0.2 (0.0-0.4)	0.1 (0.0-0.2)
Syphilis	88	0.8	1.8 (1.2-2.6)	0.0 (0.0-0.0)	0.1 (0.0-0.6)	3.0 (1.9-4.4)	0.1 (0.0-0.3)	0.0 (0.0-0.1)
*Other diseases*								
Tuberculosis	336	38	7.0 (6.5-7.5)	1.5 (0.7-2.1)	4.4 (3.5-4.6)	8.8 (7.7-9.7)	5.3 (4.5-6.9)	0.6 (0.5-0.9)
Encephalitis	179	17	3.7 (2.8-6.4)	8.8 (5.5-17.2)	2.4 (1.4-4.9)	3.9 (2.9-6.5)	2.4 (1.9-3.2)	0.6 (0.5-0.8)
Legionellosis	40	15	0.8 (0.7-1.0)	0.0 (0.0-0.0)	0.0 (0.0-0.0)	0.9 (0.7-1.1)	2.1 (1.5-3.0)	2.3 (1.8-3.2)
Creutzfeldt Jacob ^3^	9.6	7.2	0.2 (0.1-0.3)	0.0 (0.0-0.0)	0.0 (0.0-0.0)	0.1 (0.0-0.1)	1.0 (0.3-1.4)	12.3 (2.0-∞)

IDs = infectious diseases.

*Note that data for botulism, cholera, rubella and brucellosis are not presented, as no such cases aged ≥65 years occurred between 2007 and 2011 (notification rate = 0/100,000).

1: Note that food- and waterborne infections can have been transmitted in other modes than through food and water, such as via infected individuals and animals.

2: Enteropathogenic *E.*(*Escherichia*) *coli*-enteritis includes Haemolytic Uremic Syndrome.

3: For the following diseases, notification became mandatory or the last change in notification guidelines was implemented after 1993: Mumps, MRSA infections, Lyme borreliosis (1995); Creutzfeldt Jacob (1997); MRSA carriage, vancomycin-resistant enterococci, penicillin-resistant pneumococci (2005); hepatitis C (2008).

4: MRSA = Methicillin-resistant *Staphylococcus aureus.*

5: HFRS = Haemorrhagic fever with renal syndrome.

6: Because hepatitis C became notifiable only since 2008, these results only include data of 2008–2011.

7: The notification rate ratio (NRR) was calculated by dividing the average notification rate in 2007–2011 among the population ≥65 years by the average notification rate among those aged 20–64 years. A NRR above 1 reflects a higher notification rate in the population ≥65 years compared to those aged 20–64 years, while a NRR below 1 reflects a lower NRR in the population ≥65 years.

Using stratified analyses for the age-groups <5, 5–19, 20–64 and ≥65 years, we compared ID notification rates in those ≥65 years with the younger population. We calculated annual notification rate ratios (NRR) by dividing the notification rate among persons ≥65 years by the notification rate in those 20–64 years. The average annual NRR was calculated for the period 2007–2011.

We determined trends in ID notification rates from 1993 or since the year a disease became notifiable (see Footnote 3 to Table 1) and onwards, by using a non-parametric test for trend with α = 0.01. The test for trend is an extension of the Wilcoxon rank-sum test by Cuzick [16]. Only diseases with a notification rate of at least 9/100,000 in the population ≥65 years during 2007–2011 were investigated in more detail, as well as tuberculosis and VRE. This cut off was chosen based on a general conception of the burden of IDs in relation to the epidemiological situation in Norway. Tuberculosis was included because of the former high notification rate (>9/100,000 before 2000) while VRE was included due to the current high notification rate (>9/100,000 in 2011).

We determined reported place of infection, hospitalisation status, severity of infection, reason for testing, type of laboratory sample, serotype (if relevant), sex and ethnicity for IDs with an notification rate ≥9/100,000, as well as for tuberculosis and VRE (see Additional file 1). For MRSA, in addition to the division of (asymptomatic) carriage and (symptomatic) infections, we determined the median number of severe infections, defined as an infection in inner organs or a systemic infection. We classified infections into healthcare associated, community-acquired or imported, based on reported information on outbreaks in healthcare settings, work (healthcare personnel), living conditions (nursing home) and place of infection (see for more details: [17]). For VRE, we separated symptomatic infections from VRE found through screening based on the reported indication of testing. For the more in-depth analyses, we excluded years for which more than 15% of data on a specific variable were missing. Data are presented as median (range).

## Results

A total of 223,758 cases notified to MSIS between 1 January 1993 and 31 December 2011 were included in this study. Of these, 25,812 (12%) were among those ≥65 years. Infections among this population were as common in men as in women (48% male).

### Infectious disease notification rates in the population ≥65 years during 2007–2011

Generally, of all included IDs for persons ≥65 years in 2007–2011 (n = 8962), invasive bacterial diseases were most common (33% of the 65+ cases). Twenty-two percent of IDs in those ≥65 years were food- or waterborne, 21% were vaccine preventable and 12% were caused by antibiotic-resistant bacteria. Zoonoses and blood-borne diseases/STIs were least common, making up only 4.1% and 3.8%, respectively, of all cases ≥65 years.

Invasive pneumococcal disease was the most common ID among those ≥65 years during the period 2007–2011 (average annual notification rate 58/100,000; n = 411 cases on average per year), with 80% caused by serotypes included in the recommended 23-valent polysaccharide pneumococcal vaccine. Pertussis (54/100,000; n = 383) and campylobacteriosis (30/100,000; n = 217) were the 2nd and 3rd most frequently notified IDs. Other common IDs among those ≥65 years were salmonellosis (49% S. Enteritidis, 15% S. Typhimurium), MRSA infection or carriage, and invasive group B (*Streptococcus agalactiae*) or group A (*Streptococcus pyogenes*) streptococcal disease (Table 1).

### Comparison between the population ≥65 years and the younger population during 2007–2011

Although 15% of the Norwegian population is ≥65 years [15], only 11% of all notified cases from 2007 to 2011 were among those ≥65 years, resulting in an NRR of 0.73 (range 0.63-0.90). All invasive bacterial diseases, all antibiotic-resistant infections, legionellosis, listeriosis, Lyme borreliosis and prion diseases were more common in the population ≥65 years than among those aged 20–64 years (NRR > 1.0). Tetanus only occurred among those ≥65 years since 2007 (NRR = ∞), but was uncommon (on average 1 case per year). Food- and waterborne diseases (except listeriosis), blood-borne diseases/STIs, and vaccine preventable diseases were less common among persons ≥65 years (NRR < 1). Therefore, while pertussis, campylobacteriosis and salmonellosis were among the most common IDs affecting the older population, they were more common in those aged 20–64 years (NRR: 0.86, 0.41 and 0.51, respectively). We observed no consistent age patterns among the zoonoses and vector-borne diseases.

### Time trends in annual ID notification rates since 1993

**Figure 1:**
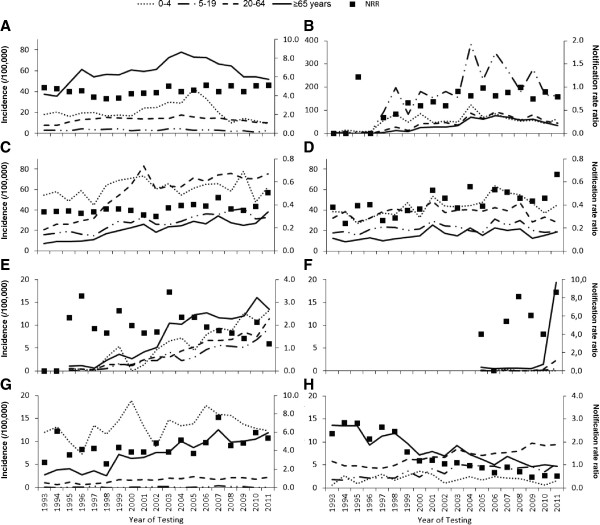
**Trends in age-specific annual notification rates (lines) and notification rate ratios (dots) of notified infectious diseases. A**: invasive pneumococcal disease, **B**: pertussis, **C**: campylobacteriosis, **D**: salmonellosis, **E**: MRSA infections, **F**: VRE*, **G**: invasive group B streptococcal disease (*Streptococcus agalactiae*), **H**: tuberculosis. Note the different Y-scales; both for the notification rates and for the notification rate ratios. *Note that VRE infections and carriage are only notifiable since 2005.

The annual notification rate in those ≥65 years remained at the same level over time for most IDs, with the exception of pertussis, campylobacteriosis, salmonellosis, invasive group B streptococcal disease, MRSA infections, tuberculosis and VRE, which changed significantly over time (Figure 1). While the test for trend was not significant for invasive pneumococcal disease (p = 0.13), the annual notification rate increased from ≤60/100,000 in the years before 2002 to 78/100,000 in 2004 but decreased thereafter to 52/100,000 in 2011 (Figure 1A). The pattern was similar for pertussis (Figure 1B); the annual notification rate increased from <30/100,000 before 2003 to 76/100,000 in 2006 and decreased subsequently to 35/100,000 in 2011.

The increase in campylobacteriosis (Figure 1C) was only observed among infections that were acquired abroad, with 34% (range 30–40) of infections among those ≥65 years in 1997–2001 being acquired abroad, compared to 44% (range 40–46) in 2007–2010. While salmonellosis is also often travel-related, the notification rate showed only a minor increase (Figure 1D) and the percentage acquired abroad remained stable (median 68% [range 58–80]).

The notification rate of MRSA infections in the 65+ population increased from 1.0/100,000 at the start of notification (1995), to 13/100,000 in 2007–2011 (Figure 1E). The increase was almost equally observed among healthcare associated infections (52% [range 39–65] of the infections), community acquired infections (24% [range 13-36]) and among infections that were acquired abroad (20% [range13-38]). The notification rate of asymptomatic MRSA carriage as well as the percentage of severe MRSA infections did not change significantly during this period (median percentage severe: 9% [range: 4.8-19]). The notification rate of VRE increased from 0.4-0.7/100,000 between 2005 and 2009 to 1.7/100,000 in 2010 and 19/100,000 in 2011 (Figure 1F). The percentage of asymptomatic carriage increased from 44% between 2005 and 2009 to more than 80% from 2010 onwards. The number of cases ≥65 years with symptoms increased from no more than 4 before 2009 to 5 in 2010 and 8 in 2011.

The notification rate of invasive group B streptococcal disease increased specifically in the population ≥65 years; the NRR changed from 4.1 in 1993–1996 to 5.6 in 2007–2011 (Figure 1G). No corresponding change over time could be observed in age at infection (78–79 years), type of laboratory sample (≥94% from blood, except in 2006: 84%), requirement for hospitalisation (>90% hospitalised) or assumed place of infection (>95% in Norway). A change specifically among the population ≥65 was also observed for tuberculosis, with the NRR decreasing from 2.5 in 1993–1996 to 0.62 in 2007–2011 (Figure 1H). While tuberculosis was common among those ≥65 years before 2000 (>9/100,000 per year), the annual notification rate decreased to 5.3/100,000 in 2007–2011. The percentage of patients ≥65 years that were born abroad increased from 12% in 1997–2001 to 25% in 2007–2011.

## Discussion

This study on notification rates of ID and trends over 19 years among the general Norwegian population ≥65 years shows that invasive bacterial diseases, and specifically invasive pneumococcal disease, were most common and occurred more often among older compared to younger individuals. Furthermore, antibiotic-resistant bacterial infections or carriage were more common in this older population and notification rates increased over time, particularly for MRSA and VRE. Overall, the population ≥65 years had less notifiable IDs than the younger population. Together with economic evaluations, including the comparison of disability adjusted life-years between diseases [18,19], this study should be used to inform public health priorities for the aging population.

Generally, comparing notification rates of IDs can be challenging because of differences in surveillance systems, populations and country-specific infection probabilities as well as changes over time in notification and diagnostic testing policies, healthcare seeking behaviour or better clinical recognition of diseases among older adults [20], which could mask changes in incidences. Our nation-wide notification data over a broad time-span allowed comparing notification rates in different IDs and age-groups over time, while taking aspects of these challenges into account. Still, the notification rate is not necessarily similar to the true incidence, as some cases never visit healthcare or are not notified. The amount of underreporting may vary between IDs and between age-groups; differential underreporting would induce bias. There are no studies available that estimate the sensitivity and representativeness of the system, but there are no indications for systematic changes in notification in recent years. The fact that the NRR did not change over time for most diseases indicated that age-specific changes in notification policy, testing policy or clinical recognition were unlikely.

Despite differences in surveillance systems across countries, our disease-specific results generally confirm observations in other countries, like the high notification rate of invasive bacterial diseases in the population ≥65 years and the increase in antibiotic-resistant bacteria in recent years [21-24]. The high notification rate of invasive bacterial diseases is likely to result from the more severe clinical presentation among older adults, rather than more transmission within this population. Older individuals are more prone to develop invasive infections due to immunosenescence [25], comorbidities, polypharmacy, and/or diminished response to antimicrobial therapy [2,4,5]. Delayed recognition of infections due to atypical presentations including the absence of fever in approximately 20% to 30% of older persons with serious infections [4,26], leading to a delayed start of therapy, is another reason why invasive disease develops more often in older individuals. As severe infections may lead to functional decline e.g. through exacerbation of underlying health problems, it is important to prevent infections and specifically severe disease in this older population. Risk communication to older people and their caregivers including encouragement to seek healthcare promptly in case of signs of systemic infection can reduce delays in starting therapy. Through vaccination against pneumococcal infections, a large portion of cases with invasive pneumococcal disease may be prevented, as 80% of infections were caused by serotypes included in the recommended 23-valent pneumococcal vaccine [27]. Although vaccination has been available in Norway for those aged ≥65 years since the 1980s, vaccine sales data indicate an uptake of only 15 to 25% (unpublished data NIPH). Prevention of invasive bacterial diseases can also be achieved through prevention of viral respiratory infections due to the increased susceptibility to bacterial super infections during an influenza infection [28,29]. It is known that the burden of influenza is highest among the older population [8,19]. We were unfortunately not able to include influenza in our analysis, because of its different surveillance system.

The overall notification rate of IDs was lower in the population ≥65 years compared to the younger population. So, while older people are at higher risk for some IDs, overall they had less notified IDs, specifically, less possible food- and waterborne diseases (except listeriosis), blood-borne diseases/STIs, and (non-invasive) vaccine preventable diseases. Different sexual behaviour, less interaction with the public [30] and reduced travel activity for the population ≥65 years compared to the younger population [31] might explain part of the lower notification rate.

The changes in notification rates over time of invasive pneumococcal disease and pertussis were likely the result of changes in vaccination policy. The decrease in invasive pneumococcal disease coincided with the introduction of pneumococcal vaccination in the childhood immunisation program in 2006 and can be explained by herd protection [32-35]. The recent decrease in pertussis notifications coincided with the introduction of a booster vaccination at 7 years of age since the school-year 2005–2006. This suggests that the booster not only protects the targeted age-groups, but also provides some protection among older adults. Still, the notification rate of pertussis remains high [36], suggesting transmission between age groups.

The increase in MRSA and VRE is worrisome, even though the notification rate in Norway is low compared to other countries [37]. It is likely that the MRSA increase resulted not only from a real increase, but also from enhanced surveillance [17]. Nevertheless, the increase in notification of symptomatic infections but not of carriage, supplemented by a time-series analysis of methicillin-sensible and -resistant cases [38], indicate also a real increase in infections. This increase is likely partly related to increased travelling and the high endemic state of MRSA in other parts of the world [17] as well as increased transmission in Norway. The increase in notified VRE since 2010 was partly due to increased screening, specifically during hospital-based outbreaks [39]. However, a real increase in VRE incidence suggested by the slight increase in the number of cases with a symptomatic VRE infection. The increases in MRSA and VRE notification rates with no change in NRR show that good compliance to infection control measures, screening of at-risk populations, and careful use of antibiotics remain important for all age-groups.

Further exploration of factors that may have caused the disease-specific changes in notification rates showed an increased notification rate of campylobacteriosis acquired in Europe. This might be the effect of increased travelling abroad [31], which increases the possibility of acquiring travel-related infections. Travel-related salmonellosis, shigellosis, giardiasis and legionellosis [40] increased much less or remained stable. These latter IDs were more often acquired outside Europe (data not shown). The difference between campylobacteriosis and the other travel-related IDs therefore may reflect more travels within Europe. Alternatively, as the change in notification rate in younger age-groups showed similar changes, the increase may reflect increased campylobacter transmission within Europe. The change in tuberculosis resulted from a decrease in notification rate among those born in Norway. This reflects the rapid decrease in tuberculosis incidence in Norway since the late 1940s [41], as tuberculosis in older adults mainly results from reactivation of previous infections, and fewer Norwegian-born people with a history of tuberculosis are still alive.

## Conclusions

Based on our results we emphasise the importance of focusing public health efforts for the ≥65 population on preventing invasive bacterial infections and antibiotic-resistant infections. Although overall the incidence of IDs among those ≥65 years was lower than in younger populations, the IDs that were more common among the population ≥65 years might be more difficult to prevent. Prevention of these IDs in the future should therefore be reinforced, as the population is aging further. Preventing bacterial infections and stopping infections from becoming invasive can be accomplished through raising awareness of the importance of vaccination in both the general population and among healthcare professionals [42], and through risk communication including encouraging older individuals and their caretakers to seek healthcare at signs of systemic infection, for instance, change of behaviour. Furthermore, improved prevention of antibiotic-resistant infections through careful use of antibiotics, good compliance to infection control measures, screening of the at-risk population, specifically in hospitals and other healthcare settings, as well as international cooperation, may prevent further increase of these infections.

## Competing interests

The authors declare that they have no competing interests.

## Authors’ contributions

AS, HME and HB made substantial contributions to the concept and design, AS analysed the data, AS, HME and HB interpreted the data, AS drafted the manuscript. All authors made important contributions to and read and approved the final manuscript.

## Pre-publication history

The pre-publication history for this paper can be accessed here:

http://www.biomedcentral.com/1471-2334/14/57/prepub

## Supplementary Material

Additional file 1 Description of the Norwegian notification system for communicable diseases with a description of the more in-depth data analyses. Click here for file

## References

[B1] Statistics Norway An aging society 2009 http://www.ssb.no/norge_en/bef_en.pdf

[B2] Gavazzi G Krause KH Ageing and infection Lancet Infect Dis 2002 14 659 666 10.1016/S1473-3099(02)00437-1 12409046

[B3] Liang SY Mackowiak PA Infections in the elderly Clin Geriatr Med 2007 14 441 456 vii 10.1016/j.cger.2007.01.010 17462528

[B4] Yoshikawa TT Epidemiology and unique aspects of aging and infectious diseases Clin Infect Dis 2000 14 931 933 10.1086/313792 10880303

[B5] Juthani-Mehta M Quagliarello VJ Infectious diseases in the nursing home setting: challenges and opportunities for clinical investigation Clin Infect Dis 2010 14 931 936 10.1086/656411 20822459 PMC3083824

[B6] Nicolle LE Strausbaugh LJ Garibaldi RA Infections and antibiotic resistance in nursing homes Clin Microbiol Rev 1996 14 1 17 8665472 10.1128/cmr.9.1.1 PMC172878

[B7] Norwegian Institute of Public Health **Causes of death from 1951–2004** [In Norwegian] 2012 http://www.fhi.no/tema/dodsaarsaker-oglevealder/dodsaarsaker-1951-2004

[B8] Jefferson T Di Pietrantonj C Al Ansary LA Ferroni E Thorning S Thomas RE Vaccines for preventing influenza in the elderly Cochrane Database Syst Rev 2010 14 CD004876 20166072 10.1002/14651858.CD004876.pub3

[B9] Nicolle LE Urinary tract infection in long-term-care facility residents Clin Infect Dis 2000 14 757 761 10.1086/313996 11017826

[B10] Choi C Bacterial meningitis in aging adults Clin Infect Dis 2001 14 1380 1385 10.1086/322688 11550119

[B11] Edwards MS Baker CJ Group B streptococcal infections in elderly adults Clin Infect Dis 2005 14 839 847 10.1086/432804 16107984

[B12] Kirk MD Gregory J Lalor K Hall GV Becker N Foodborne and waterborne infections in elderly community and long-term care facility residents, Victoria, Autralia Emerg Infect Dis 2012 14 377 384 10.3201/eid1803.110311 22377177 PMC3309568

[B13] Chami K Gavazzi G Carrat F de Wazières B Lejeune B Piette F Rothan-Tondeur M Burden of infections among 44,869 elderly in nursing homes: a cross-sectional cluster nationwide survey J Hosp Infect 2011 14 254 259 10.1016/j.jhin.2011.08.003 21899920

[B14] Norwegian Surveillance System for Communicable Diseases (MSIS) http://www.msis.no/

[B15] Statistics Norway Population at 1st of January in 1990 up to 2011 2011 http://www.ssb.no/a/aarbok/tab/tab-048.html

[B16] Nonparametric test for trend across ordered groups http://www.stata.com/manuals13/rnptrend.pdf

[B17] Elstrøm P Kacelnik O Bruun T Iversen B Hauge SH Aavitsland P Meticillin-resistant Staphylococcus aureus in Norway, a low-incidence country, 2006–2010 J Hosp Infect 2012 14 36 40 10.1016/j.jhin.2011.10.004 22118858

[B18] European Centre for Disease Prevention and Control Current and future burden of communicable diseases in the European Union and EEA/EFTA countries - Methodology protocol http://www.ecdc.europa.eu/en/publications/publications/1106_ter_burden_of_disease.pdf

[B19] Health statistics and health information systems. Global burden of disease (GBD) 2001 estimates http://www.who.int/healthinfo/global_burden_disease/estimates_regional_2001/en/

[B20] High KP Bradley SF Gravenstein S Mehr DR Quagliarello VJ Richards C Yoshikawa TT Clinical practice guideline for the evaluation of fever and infection in older adult residents of long-term care facilities: 2008 update by the Infectious Diseases Society of America J Am Geriatr Soc 2009 14 375 394 10.1111/j.1532-5415.2009.02175.x 19278394 PMC7166905

[B21] European Centre of Disease Prevention and Control Annual epidemiological report on communicable diseases in Europe 2010 http://www.ecdc.europa.eu/en/publications/Publications/1011_SUR_Annual_Epidemiological_Report_on_Communicable_Diseases_in_Europe.pdf 22114980

[B22] Dworkin MS Park L Borchardt SM The changing epidemiology of invasive Haemophilus influenzae disease, especially in persons > or = 65 years old Clin Infect Dis 2007 14 810 816 10.1086/511861 17304452

[B23] Rubach MP Bender JM Mottice S Hanson K Weng HY Korgenski K Daly JA Pavia AT Increasing incidence of invasive Haemophilus influenzae disease in adults, Utah, USA Emerg Infect Dis 2011 14 1645 1650 10.3201/eid1709.101991 21888789 PMC3322072

[B24] Lambertsen L Ekelund K Skovsted IC Liboriussen A Slotved HC Characterisation of invasive group B streptococci from adults in Denmark 1999 to 2004 Eur J Clin Microbiol Infect Dis 2010 14 1071 1077 10.1007/s10096-010-0941-z 20676713

[B25] Castle SC Clinical relevance of age-related immune dysfunction Clin Infect Dis 2000 14 578 585 10.1086/313947 10987724

[B26] Wester AL Dunlop O Melby KK Dahle UR Wyller TB Age-related differences in symptoms, diagnosis and prognosis of bacteremia BMC Infect Dis 2013 14 346 10.1186/1471-2334-13-346 23883345 PMC3733624

[B27] Pneumovax® 23 (pneumococcal vaccine polyvalent) http://www.merck.com/product/usa/pi_circulars/p/pneumovax_23/pneumovax_pi.pdf

[B28] Palacios G Hornig M Cisterna D Savji N Bussetti AV Kapoor V Hui J Tokarz R Briese T Baumeister E Streptococcus pneumoniae coinfection is correlated with the severity of H1N1 pandemic influenza PLoS One 2009 14 e8540 10.1371/journal.pone.0008540 20046873 PMC2795195

[B29] O’Brien KL Walters MI Sellman J Quinlisk P Regnery H Schwartz B Dowell SF Severe pneumococcal pneumonia in previously healthy children: the role of preceding influenza infection Clin Infect Dis 2000 14 784 789 10.1086/313772 10816149

[B30] Mossong J Hens N Jit M Beutels P Auranen K Mikolajczyk R Massari M Salmaso S Tomba GS Wallinga J Social contacts and mixing patterns relevant to the spread of infectious diseases PLoS Med 2008 14 e74 10.1371/journal.pmed.0050074 18366252 PMC2270306

[B31] Statistics Norway Travel survey https://www.ssb.no/statistikkbanken/SelectVarVal/Define.asp?MainTable=Turer2&KortNavnWeb=reise&PLanguage=0&checked=true

[B32] Lexau CA Lynfield R Danila R Pilishvili T Facklam R Farley MM Harrison LH Schaffner W Reingold A Bennett NM Changing epidemiology of invasive pneumococcal disease among older adults in the era of pediatric pneumococcal conjugate vaccine JAMA 2005 14 2043 2051 10.1001/jama.294.16.2043 16249418

[B33] Hammitt LL Bruden DL Butler JC Baggett HC Hurlburt DA Reasonover A Hennessy TW Indirect effect of conjugate vaccine on adult carriage of Streptococcus pneumoniae: an explanation of trends in invasive pneumococcal disease J Infect Dis 2006 14 1487 1494 10.1086/503805 16652275

[B34] Miller E Andrews NJ Waight PA Slack MP George RC Herd immunity and serotype replacement 4 years after seven-valent pneumococcal conjugate vaccination in England and Wales: an observational cohort study Lancet Infect Dis 2011 14 760 768 10.1016/S1473-3099(11)70090-1 21621466

[B35] Steens A Bergsaker MA Aaberge IS Rønning K Vestrheim DF Prompt effect of replacing the 7-valent pneumococcal conjugate vaccine with the 13-valent vaccine on the epidemiology of invasive pneumococcal disease in Norway Vaccine 2013 14 6232 6238 24176490 10.1016/j.vaccine.2013.10.032

[B36] EUvac.net Pertussis surveillance report 2010 2011 http://www.ecdc.europa.eu/en/publications/Publications/pertussis_report_2010_euvacnet.pdf

[B37] Kalenic S Cookson B Gallagher R Popp W Asensio-Vegas A Assadian O Blok O Brussafero S Eastaway A Elstrom P Comparison of recommendations in national/regional Guidelines for prevention and control of MRSA in thirteen European countries Int J Infection Control 2010 14 doi:10.3396/ijic.V6i2.016.10

[B38] Moxnes JF de Blasio BF Leegaard TM Moen AE Methicillin-resistant Staphylococcus aureus (MRSA) is increasing in Norway: a time series analysis of reported MRSA and methicillin-sensitive S. aureus cases, 1997–2010 PLoS One 2013 14 e70499 10.1371/journal.pone.0070499 23936442 PMC3731260

[B39] Kacelnik O Ward based cohort study of the first reported outbreak of Vancomycin resistant enterococci in a Norwegian hospital [abstract] Eur Sci Conference Appl Infectious Disease Epidemiology 2012 Abstract number 2012663

[B40] Guzman-Herrador B Vold L Nygård K Surveillance of travel-associated gastrointestinal ionfections in Norway, 2009–2010: are they actually imported? Eurosurveillance 2012 14 41 http://www.eurosurveillance.org/ViewArticle.aspx?ArticleId=20294 23078812

[B41] Heldal E Docker H Caugant DA Tverdal A Pulmonary tuberculosis in Norwegian patients. The role of reactivation, re-infection and primary infection assessed by previous mass screening data and restriction fragment length polymorphism analysis Int J Tuberc Lung Dis 2000 14 300 307 10777077

[B42] Michel JP Updated vaccine guidelines for aging and aged citizens of Europe Expert Rev Vaccines 2010 14 7 10 10.1586/erv.10.27 20192711

