# Supplementary material for: What are the most important infectious diseases among those ≥65 years: a comprehensive analysis on notifiable diseases, Norway, 1993–2011
Source: BMC Infect Dis. 2014 Feb 4;14:57. doi: 10.1186/1471-2334-14-57 (PMC3923236; doi:10.1186/1471-2334-14-57)
Supplement: Additional file 1 — Description of the Norwegian notification system for communicable diseases with a description of the more in-depth data analyses. [file 1471-2334-14-57-S1.docx]

**Supplementary Material belonging to “***What are the most important infectious diseases among those ≥65 years: A comprehensive analysis on notifiable diseases, Norway, 1993-2011***”**

**Description of the Norwegian notification system for communicable diseases**

The infectious diseases that are notifiable to the Norwegian Surveillance System for Communicable Diseases (MSIS) are classified in 3 groups (see Table S1). For diseases of category A, full patient information is available including the personal identification number that each inhabitant receives at birth or when immigrated to Norway. The identification number is used to match reports from laboratories and clinicians. Cases of category B diseases are notified without name or personal identification number. Laboratory and clinical data are linked using a numbered notification form that the laboratory sends to both the clinician and the NIPH and which the clinician subsequently fills out and sends to the NIPH. Data on diseases in category C are reported by laboratories with limited descriptive information, and no identifiable data. Note that a case can suffer from different infections at the same time.

*Table S1: Categorisation of the notifiable diseases in Norway.*

| Group A | | Group B | Group C |
| --- | --- | --- | --- |
| Anthrax | Mumps | Gonorrhoea | Chlamydia |
| AIDS | HFRS^2^ / Nephropathia epidemica | HIV infection | Influenza |
| Botulism | Paratyphoid fever | Syphilis |  |
| Brucellosis | Pertussis |  |  |
| Campylobacteriosis | Plague |  |  |
| Cholera | Pneumococcal invasive disease |  |  |
| Creutzfeldt Jacob | Poliomyelitis |  |  |
| Diphtheria | PRP infections and carrier states ^3^ |  |  |
| Echinococcal disease | Rabies |  |  |
| Encephalitis | Relapsing fevers |  |  |
| Enteropathogenic *Escherichia coli*-enteritis | Rubella |  |  |
| Giardiasis | Salmonellosis |  |  |
| Haemolytic Uremic Syndrome | Severe Acute Respiratory Syndrome |  |  |
| Haemorrhagic fevers | Shigellosis |  |  |
| *Haemophilus influenzae* invasive disease | Smallpox |  |  |
| Hepatitis A | Streptococcal invasive disease, group A |  |  |
| Hepatitis B acute infection and carriage | Streptococcal invasive disease, group B |  |  |
| Hepatitis C | Tetanus |  |  |
| Influenza A (H1N1) | Trichinosis |  |  |
| Legionellosis | Tuberculosis |  |  |
| Leprosy | Tularaemia |  |  |
| Listeriosis | Typhus (epidemic) |  |  |
| Lyme borreliosis | Typhoid fever |  |  |
| Malaria | VRE infection and carriage ^4^ |  |  |
| Measles | Yellow fever |  |  |
| Meningococcal invasive disease | Yersiniosis |  |  |
| MRSA infection and carriage ^1^ |  |  |  |

1 MRSA = Methicillin-resistant Staphylococcus aureus

2 Haemorrhagic fever with renal syndrome

3 PRP = Penicillin-resistant pneumococci

4 VRE = Vancomycin-resistant enterococci

**Data analysis**

The following more in-depth, disease specific investigations were performed: for invasive pneumococcal disease we determined the vaccine-specific serotypes for the period 2007-2011. We excluded cases whose serotype was unavailable (2.7%). For campylobacteriosis, salmonellosis, and invasive group A and group B streptococcal disease we determined the assumed place of infection (in Norway or abroad). For invasive group A and group B streptococcal disease we also determined the average age of the cases ≥65 years per time period, the requirement for hospitalisation and kind of laboratory sample. For MRSA infections we determined the severity of the infection and the percentage of cases being health-care associated or community required. Due to slight changes in notification requirements of the latter variables, we only analysed data from 2004 onwards. Tuberculosis was analysed by the ethnicity of the cases (Norwegian / non-Norwegian). For the sub-analyses on changes over time we excluded years for which more than 15% of data on the specific variable were missing (for assumed place of infection: 1993, 1994 and 2011 for campylobacteriosis and salmonellosis, and all years before 2007 for invasive group A and group B streptococcal disease; for ethnicity: 1993-1995 for tuberculosis; severity of MRSA infection, health-care associated and community acquired MRSA: before 1999 and 2011).
